# Prevalence and determinants of inappropriate endotracheal cuff pressure in adult surgical patients undergoing general anesthesia: a prospective observational study

**DOI:** 10.1186/s12871-026-03738-7

**Published:** 2026-03-12

**Authors:** Nuanprae Kitisin, Arada Sanggasameson, Chatwatsaya Suphasirichatthana, Chawanlak Prajongmool, Surapa Tornsatitkul, Nattaya Raykateeraroj

**Affiliations:** Department of Anesthesiology, Faculty of Medicine Siriraj Hospital, Mahidol University, 2 Wanglang Road, Bangkok, 10170 Thailand

**Keywords:** Airway safety, Endotracheal cuff, Intraoperative monitoring, Patient safety, Risk management, Tracheal injury

## Abstract

**Background:**

Endotracheal tube (ETT) cuff pressure should be maintained between 20 – 30 cmH₂O to prevent air leak, aspiration, and tracheal injury. Cuff inflation is frequently guided by clinical judgement rather than manometry. Data on inappropriate cuff pressure prevalence and determinants in Asian surgical populations are limited.

**Methods:**

We prospectively studied adult patients undergoing elective surgery with endotracheal intubation at a tertiary hospital. After intubation, anaesthesia providers inflated the ETT cuff per usual practice based on clinical judgement without manometry. The research team measured initial cuff pressure with a manometer and adjusted it to 20 – 30 cmH₂O, recording initial and final pressures and volumes. The primary outcome was prevalence of inappropriate initial cuff pressure (< 20 or > 30 cmH₂O). Secondary analyses were exploratory, using multinomial logistic regression to examine associated factors and models for cuff inflation volume and final pressure.

**Results:**

Of 270 patients, only 107 (39.6%) had initial cuff pressure within the recommended range; 77 (28.5%) were < 20 cmH₂O and 86 (31.9%) were > 30 cmH₂O. Patient characteristics (age, sex, body mass index) were not associated with cuff-pressure category. ETT brand showed significant associations with excessive pressure. Postoperative airway symptoms were infrequent and similar across groups. An exploratory multivariable model incorporating age, sex, ETT size, and brand demonstrated moderate explanatory performance for cuff inflation volume (coefficient of determination [R^2^] = 0.336).

**Conclusion:**

Empiric cuff inflation results in inappropriate ETT cuff pressure in nearly two-thirds of adult surgical patients, and common clinical variables do not reliably predict safe pressure. Routine manometric monitoring should be used to ensure adequate cuff inflation; reliance on fixed inflation volumes or simple volume-based formulas is not recommended.

**Trial registration:**

Not applicable.

**Supplementary Information:**

The online version contains supplementary material available at 10.1186/s12871-026-03738-7.

## Background

Endotracheal tubes (ETT) serve a critical role in protecting the airway from aspiration and enabling positive pressure ventilation during anesthesia and critical care. Modern ETTs are typically constructed from polyvinyl chloride (PVC) and feature a thin, flexible, high-volume, low-pressure cuff near the distal end. This cuff, when inflated, creates a seal against the tracheal wall [1, 2]. Proper cuff inflation prevents aspiration of oropharyngeal secretions and ensures effective ventilation. The optimal cuff pressure, measured via the pilot balloon and expressed in centimeters of water (cmH₂O), ranges between 20 and 30 cmH₂O [3–5].

The consequences of deviating from this range can be significant. Cuff pressures below 20 cmH₂O may allow air leakage, aspiration of secretions, or loss of anesthetic gases. Conversely, pressures exceeding 30 cmH₂O risk compromising tracheal mucosal perfusion, potentially leading to ischemic injury, ulceration, stenosis, or in rare cases, tracheal rupture [1, 6–15]. The clinical burden of these complications is considerable: postoperative sore throat affects up to 61.8% of patients following general anesthesia [16], while recent ICU cohorts report tracheal stenosis in around 8–9% of patients after prolonged intubation or tracheostomy [17].

Despite clear guidelines, maintaining appropriate cuff pressure remains problematic in clinical practice. Multicenter studies consistently reveal that only 10–30% of intubated surgical patients have cuff pressures within the recommended range, with most being either over- or underinflated [3, 18–20]. Liu et al. [20] demonstrated that inappropriate cuff pressure monitoring correlates with higher rates of postoperative airway complications, including cough and sore throat. Similarly, Sengupta et al. [3] found that half of their patients had cuff pressures exceeding 30 cmH₂O, while both Gilliland et al. and Orandi et al. [18, 19] reported mean pressures well above safe limits, particularly in operating room environments. Recent reviews confirm that reliance on clinical estimation rather than objective manometric measurement continues to be widespread, especially in busy operating suites where simultaneous case starts may limit access to cuff pressure manometers [4, 21].

Although cuff pressure monitoring is recognized as a patient safety standard, routine measurement is still infrequent worldwide. In practice, anesthetists often inflate the cuff empirically—typically with 5–10 mL of air depending on patient and ETT characteristics—without verifying the actual pressure. This pragmatic approach, though convenient, risks both under- and over-inflation, which may compromise airway safety.

The present study was designed to address these gaps. Accordingly, the primary objective was to estimate the prevalence of inappropriate endotracheal cuff pressure in adult patients undergoing elective surgery under general anesthesia. Secondary objectives were exploratory and included describing patient- and device-related factors associated with pressure deviations and evaluating whether routinely available clinical variables could meaningfully inform cuff inflation volume and achieved cuff pressure. By clarifying the limited utility of empirical estimation strategies in real-world operating environments where manometric monitoring is not routinely used, this study aims to provide contemporary data to support safer cuff-management practices.

## Methods

### Study design and ethical approval

This prospective observational study was conducted after approval from the Siriraj Institutional Review Board (COA Si 350/2024) and in accordance with the Declaration of Helsinki and institutional research guidelines. Written informed consent was obtained from all participants prior to enrollment. This report follows the Strengthening the Reporting of Observational Studies in Epidemiology (STROBE) guidelines for observational studies [22] (Supplementary Appendix I).

### Participants

Adult patients aged 18 years or older who were scheduled for elective surgery under general anesthesia with endotracheal intubation and an expected anesthesia duration exceeding one hour were consecutively recruited. Patients with known anatomical laryngotracheal abnormalities, known or anticipated difficult intubation, or pregnancy were excluded. All eligible participants were enrolled in a single observational cohort, and no randomization or group allocation was performed.

### Anesthetic management

General anesthesia was induced using standard intravenous agents, and neuromuscular blockade was achieved with either succinylcholine or a non-depolarizing muscle relaxant. Male patients were routinely intubated with a tube of 7.5 – 8.0 mm internal diameter, and female patients with 7.0 – 7.5 mm, consistent with institutional practice. Anesthesia was maintained with volatile agents in a mixture of oxygen and air; nitrous oxide was not used. All ETTs were high-volume, low-pressure polyvinyl chloride types. Three commercially available brands were used: Covidien™, Portex™, and Shaoxing™. In addition to demographic and procedural characteristics, airway pressure parameters were routinely documented as part of intraoperative monitoring. These included peak inspiratory pressure (PIP) and positive end-expiratory pressure (PEEP), which were incorporated into subsequent exploratory analyses.

### Cuff pressure measurement protocol

After endotracheal intubation and stabilization of vital signs, cuff pressure was measured with the patient in the supine position using a Hi-Lo™ Hand Pressure Gauge (Covidien, USA) connected directly to the pilot balloon via a flexible connecting tube. According to the manufacturer’s instructions, the device could be attached directly without a three-way stopcock. Measurements were taken at end-expiration and recorded in cmH₂O.

All intubations and cuff inflations were performed by the attending anesthesia physicians or anesthesia residents as part of routine clinical practice. The intubator or circulating anaesthesia nurse inflated the cuff based on their clinical judgment, and the initial inflation volume was recorded before the research team performed any measurement. The research team was not involved in intubation and was blinded to the provider's clinical assessment. However, blinding to measured pressure was not feasible during adjustments. To minimize bias, all measurements followed a standardized written protocol.

If the measured pressure was within 20 – 30 cmH₂O, no adjustment was made. When pressure exceeded 30 cmH₂O, air was withdrawn from the cuff in 0.5–1 mL increments, with pressure rechecked after each adjustment until it fell below 30 cmH₂O. The total air volume removed was recorded and subtracted from the initial inflation volume to determine the final cuff volume. Conversely, when pressure was below 20 cmH₂O, air was added in 0.5 – 1 mL increments until it exceeded 20 cmH₂O. The total air volume added was recorded and combined with the initial volume to calculate the final cuff volume. If an audible air leak persisted despite achieving a pressure within 20 – 30 cmH₂O, additional air (0.5 – 1 mL at a time) was injected until the leak resolved, and cuff pressure was rechecked. If an adequate seal could not be achieved without exceeding 30 cmH₂O, a larger tube size was recommended to the attending anesthesiologist.

For analysis, the following parameters were defined:

- Initial cuff volume (mL): the volume of air initially used to inflate the ETT cuff by the anesthesia provider before any measurement or adjustment.
- Initial cuff pressure (cmH₂O): the first measured cuff pressure immediately after intubation and stabilization of vital signs.
- Adjusted cuff volume (mL): the final volume of air in the cuff after adjustment to achieve a target pressure within 20 – 30 cmH₂O.
- Adjusted cuff pressure (cmH₂O): the final cuff pressure recorded after adjustment.
- ΔCuff pressure (cmH₂O): the change in cuff pressure from pre- to post-adjustment, calculated as Adjusted cuff pressure – Initial cuff pressure.

### Outcomes

The primary outcome was the prevalence of inappropriate endotracheal cuff pressure. An initial cuff pressure between 20 and 30 cmH₂O was defined as adequate, whereas values outside this range were considered inappropriate, classified as “Too-low” (< 20 cmH₂O) or “Too-high” (> 30 cmH₂O).

Secondary outcomes were exploratory and included comparisons of baseline demographic and procedural characteristics across cuff-pressure categories; pre- and post-adjustment cuff parameters (initial/adjusted pressure and volume, and intraoperative airway pressures); postoperative airway complications; factors associated with inappropriate cuff pressure; the relationship between cuff inflation volume and achieved pressure; and the performance of prediction models for cuff inflation volume and final cuff pressure.

Postoperative airway complications were assessed 24 h after surgery using a standardized questionnaire administered during routine postoperative rounds. Evaluated complications included sore throat (patient-reported discomfort or pain in the throat at rest or during swallowing), hoarseness (patient-perceived change in voice quality relative to preoperative baseline), and blood-stained sputum (visible blood streaks in expectorated secretions). Each event was documented as present or absent based on patient report with clinical confirmation.

### Statistical analysis

Sample size was determined from Gilliland et al. [18], which reported an optimal-range cuff pressure incidence of 18.75%. Assuming an expected proportion of 20%, a 5% margin of error, and a 95% confidence level, 246 patients were required. Allowing for 10% incomplete data gave a target sample size of 270.

All analyses were conducted using R software (version 4.4.3). No variables had missing data; all 270 enrolled patients contributed complete information for the planned analyses. Consequently, all analyses were performed on 270 complete cases and no imputation was required. Analyses consisted of: (i) descriptive estimation of the prevalence of inappropriate cuff pressure (primary analysis); and (ii) secondary exploratory analyses, including comparisons across cuff-pressure categories, evaluation of patient- and device-related factors associated with pressure deviations, and the development of prediction models for cuff inflation volume and final cuff pressure.

Distributional characteristics of continuous variables were inspected using histograms, quantile–quantile plots, and the Shapiro–Wilk test. Normally distributed variables were summarized as mean ± standard deviation (SD) and compared using one-way analysis of variance. Non-normal variables were summarized as median with interquartile range and compared using the Kruskal–Wallis test with Bonferroni-adjusted pairwise Wilcoxon rank-sum tests. Effect sizes were quantified using eta-squared for continuous variables and Cramer’s V for categorical variables.

Multinomial logistic regression was used in exploratory analyses to examine factors associated with inappropriate cuff pressure (Too-low or Too-high vs. adequate). Candidate predictors included age, sex, BMI, operation category, ETT size, and ETT brand. Univariable screening guided multivariable model inclusion, with clinically relevant variables retained regardless of *P*-value. Odds ratios with 95% confidence intervals were reported.

For multi-level categorical predictors in multinomial regression models (including operation category and ETT brand), global P-values were derived from likelihood ratio tests and subsequently adjusted for multiple comparisons using the Benjamini–Hochberg false discovery rate (FDR) method. Bonferroni correction was applied only to pairwise post hoc comparisons following significant omnibus tests in univariable analyses. This approach was selected to balance control of type I error in exploratory multivariable modelling while avoiding excessive conservatism in limited pairwise comparisons.

For prediction modelling, multivariable linear regression was used in exploratory analyses to estimate the cuff inflation volume required to achieve an adequate pressure of 20 – 30 cmH₂O. Predictors included age, sex, BMI, ETT size, and ETT brand. Model performance was evaluated using R^2^, root-mean-square error, and mean absolute error, with internal validation by repeated ten-fold cross-validation.

A second exploratory model was developed to examine the relationship between cuff inflation volume and achieved cuff pressure. Both linear regression and generalized additive models (GAMs) with penalized splines were explored to account for potential nonlinear relationships. Model comparison was performed using likelihood-ratio tests, Akaike information criterion, and Bayesian information criterion. Agreement between predicted and observed values was assessed using scatter plots and Bland–Altman analysis. Model assumptions (linearity, normality of residuals, homoscedasticity, and influential outliers) were inspected using standard diagnostic plots. A two-sided *P*-value < 0.05 was considered statistically significant. Given the exploratory nature of the secondary analyses, effect sizes and overall patterns were emphasized over statistical significance alone.

## Results

### Prevalence of inappropriate cuff pressure

**Fig. 1:**
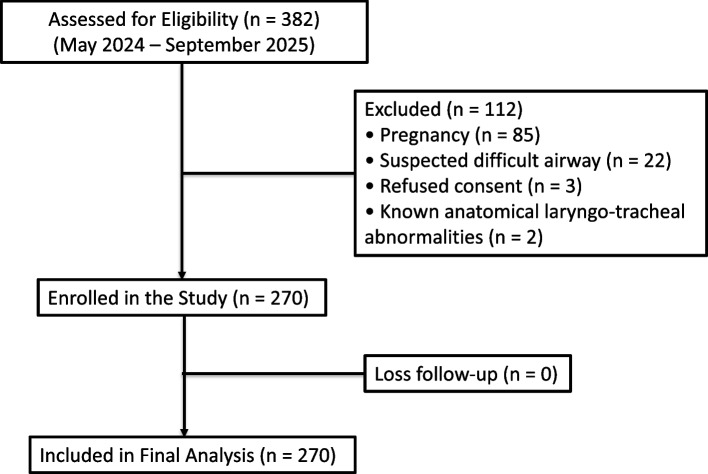
Study flow. Between May 2024 and September 2025, 382 patients were assessed for eligibility; 112 were excluded (85 due to pregnancy, 22 with suspected difficult airway, 3 refused consent, and 2 with known anatomical laryngotracheal abnormalities), leaving 270 patients who were enrolled and included in the final analysis

Between 23 May 2024 and 23 September 2025, 382 patients were assessed for eligibility. Of these, 112 were excluded (85 due to pregnancy, 22 with suspected difficult airway, 3 declined consent, and 2 with known anatomical laryngotracheal abnormalities), resulting in 270 patients who were enrolled and analysed (Fig. 1). Only 107 patients (39.6%) had cuff pressure within the recommended range of 20 – 30 cmH₂O, while 163 (60.4%) exhibited inappropriate pressure. Among these, 77 patients (28.5%) had cuff pressure < 20 cmH₂O (“Too-low”), and 86 patients (31.9%) had > 30 cmH₂O (“Too-high”).

### Baseline characteristics and pre-adjustment parameters

Baseline demographic and procedural characteristics were broadly comparable across pressure groups (Table 1). Age, sex distribution, and body mass index did not differ significantly. However, the distribution of operation categories and ETT brand varied across groups (global *P* = 0.023 and 0.025, respectively).

**Table 1:** Baseline characteristics and pre-adjustment cuff parameters by pressure group

**Variables**	**Adequate cuff pressure (*n =* 107)**	**Inappropriate cuff pressure (*n =* 163)**		***P*-value**	**Effect size**^††^
		**Too-low (*n =* 77)**	**Too-high (*n =* 86)**		
Age, years	62.0 (47.0, 69.0)	63.0 (46.0, 71.0)	59.0 (41.2, 69.0)	0.226	0.011
BMI, kg/m^2^	24.2 (21.9, 26.6)	23.3 (21.1, 25.4)	24.2 (20.0, 28.2)	0.356	0.008
Male	45 (42.1%)	40 (51.9%)	33 (38.4%)	0.198	0.110
Operation				0.023^*^	0.198
Eye-ENT surgery	6 (5.6%)	6 (7.8%)	7 (8.1%)		
Head and neck surgery	11 (10.3%)	9 (11.7%)	5 (5.8%)		
Abdominal surgery	60 (56.1%)	41 (53.2%)	31 (36.0%)		
Urogynecologic surgery	15 (14.0%)	8 (10.4%)	12 (14%)		
Orthopedic surgery	12 (11.2%)	9 (11.7%)	18 (20.9%)		
Other surgery^†^	3 (2.8%)	4 (5.2%)	13 (15.1%)		
ETT brand				0.025^*^	0.143
Covidien	16 (15.0%)	15 (19.5%)	23 (26.7%)		
Portex	19 (17.8%)	19 (24.7%)	7 (8.1%)		
Shaoxing	72 (67.3%)	43 (55.8%)	56 (65.1%)		
ETT size				0.300	0.107
6.5	1 (0.9%)	0 (0.0%)	1 (1.2%)		
7.0	51 (47.7%)	33 (42.9%)	43 (50.0%)		
7.5	26 (24.3%)	24 (31.2%)	29 (33.7%)		
8.0	29 (27.1%)	20 (26.0%)	13 (15.1%)		
Pre-adjustment ETT cuff parameters					
Initial cuff volume, mL	5.0 (5.0, 6.0)	5.0 (5.0, 6.0)	6.0 (5.0, 6.0)	< 0.001^*^	0.054
Initial cuff pressure, cmH_2_O	25.0 (22.0, 27.0)	14.0 (10.0, 18.0)	42.0 (38.0, 60.0)	< 0.001^*^	0.884
Pre-adjustment ventilation detail					
PIP, cmH_2_O	19.0 (16.0, 20.5)	18.0 (16.0, 20.0)	19.5 (16.0, 21.0)	0.047*	0.023
PEEP, cmH_2_O	5.0 (5.0, 5.0)	5.0 (5.0, 5.0)	5.0 (5.0, 5.0)	0.660	0.003

Data are presented as median (IQR, interquartile range) for continuous variables and number (%) for categorical variables. P-values were calculated using the Kruskal–Wallis test for continuous variables and the χ^2^ test or Fisher’s exact test for categorical variables, as appropriate

*ETT* Endotracheal tube, *BMI* Body mass index, *ENT* Ear, nose, and throat, *PIP* Peak inspiratory pressure, *PEEP* Positive end-expiratory pressure

^†^Other surgery comprises radiologic, neurosurgical, endoscopic, and plastic procedures

^††^Effect size: η^2^ for continuous variables (Kruskal–Wallis) and Cramer’s V for categorical variables. Benchmarks: small = 0.01/0.1, medium = 0.06/0.3, large = 0.14/0.5, respectively. No missing data were present for the reported variables; all analyses were performed on 270 complete cases

^*^P-value < 0.05 was considered statistically significant

**Fig. 2:**
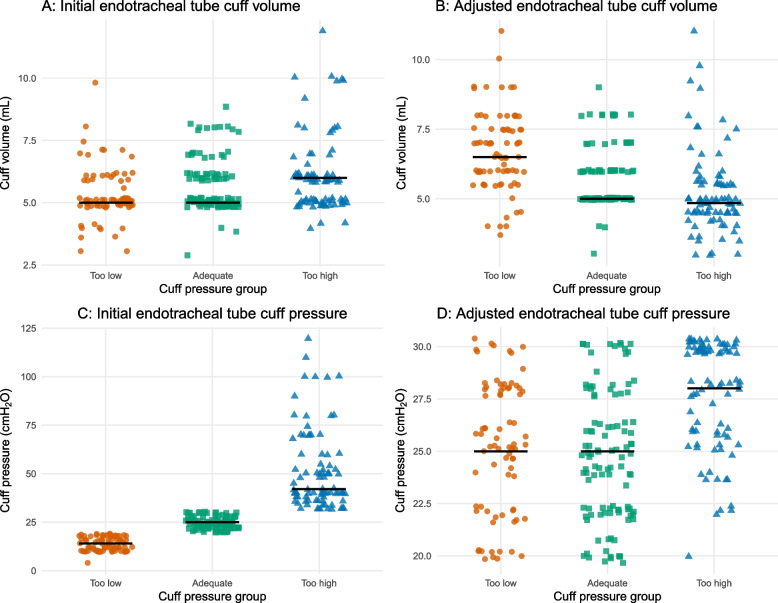
Pre- and post-adjustment endotracheal tube cuff parameters by pressure group. **A** Cuff volume before adjustment, **(B)** cuff volume after adjustment, **(C)** cuff pressure before adjustment, and **(D)** cuff pressure after adjustment across the Too-low (< 20 cmH₂O), Adequate (20 – 30 cmH₂O), and Too-high (> 30 cmH₂O) pressure groups. Each dot represents an individual measurement, with black horizontal bars indicating median values. Statistical comparisons were performed using pairwise Wilcoxon rank-sum tests with Bonferroni adjustment (see Supplementary Table S1)

Pre-adjustment cuff parameters demonstrated marked differences: patients in the Too-low group showed substantially lower initial cuff pressure, while those in the Too-high group had distinctly elevated pressures (all pairwise *P* < 0.001). Initial cuff volume differed modestly between groups. Pre-adjustment peak inspiratory pressure was slightly lower in the Too-low group (*P* = 0.047), whereas positive end-expiratory pressure was similar across groups. Figure 2 illustrates pre- and post-adjustment cuff volumes and pressures across the three groups.

### Post-adjustment cuff parameters and adverse events

**Fig. 3:**
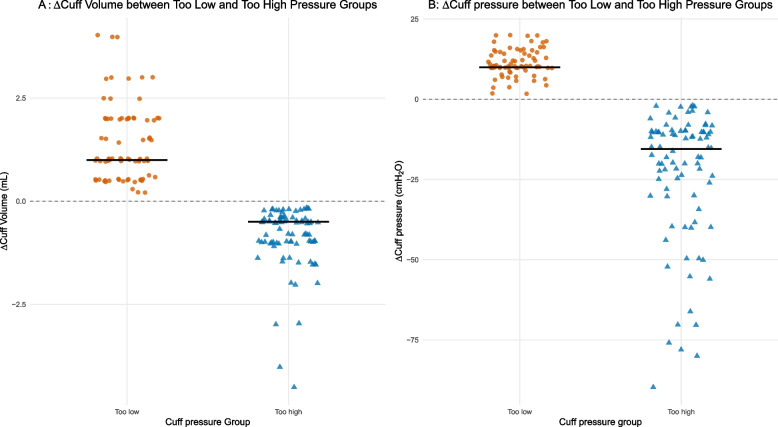
Changes in cuff volume and pressure between the Too-low and Too-high pressure groups. **A** ΔCuff volume and (**B**) ΔCuff pressure represent changes from pre- to post-adjustment values. Each point represents an individual patient; black bars indicate group medians. Positive Δ values indicate an increase after adjustment, whereas negative Δ values indicate a decrease. Cuff volume increased in the Too-low group and decreased in the Too-high group, corresponding to increases and reductions in cuff pressure, respectively (pairwise Wilcoxon rank-sum tests with Bonferroni adjustment; all P < 0.001)

After standardized adjustment to a target cuff pressure of 20 – 30 cmH₂O, cuff pressures became comparable between the Too-low and Adequate groups, although the Too-high group remained slightly but significantly higher (Table 2). Adjusted cuff volume remained differ across groups (*P* < 0.001), with the Too-low group requiring the largest volume increase and the Too-high group requiring substantial air removal, consistent with the observed changes in cuff pressure (Fig. 3).

**Table 2:** Post-adjustment cuff parameters and related outcomes

**Variables**	**Adequate cuff pressure (*n =* 107)**	**Inappropriate cuff pressure (*n =* 163)**		***P*-value**	**Effect size**^†^
		**Too-low (*n =* 77)**	**Too-high (*n =* 86)**		
Intubation attempt				0.347	0.093
1	105 (98.1%)	73 (94.8%)	85 (98.8%)		
2	1 (0.9%)	1 (1.3%)	1 (1.2%)		
3	1 (0.9%)	3 (3.9%)	0 (0.0%)		
Post-adjustment ETT cuff parameters					
Adjusted cuff volume, mL	5.0 (5.0, 6.0)	6.5 (6.0, 7.5)	4.9 (4.5, 5.6)	< 0.001^*^	0.220
Adjusted cuff pressure, cmH_2_O	25.0 (22.0, 27.0)	25.0 (22.0, 28.0)	28.0 (26.0, 30.0)	< 0.001^*^	0.178
Adverse event					
Sore throat	28 (26.2%)	17 (22.1%)	22 (25.6%)	0.975	0.030
Hoarseness	10 (9.3%)	7 (9.1%)	8 (9.3%)	> 0.999	0.011
Blood in sputum	0 (0.0%)	2 (2.6%)	0 (0.0%)	0.276	0.097

Data are presented as median (IQR, interquartile range) for continuous variables and number (%) for categorical variables. *P*-values were calculated using the Kruskal–Wallis test for continuous variables and the χ^2^ test or Fisher’s exact test for categorical variables, as appropriate

*ETT* Endotracheal tube, *cmH₂O* centimeters of water

^†^Effect size: η^2^ for continuous variables (Kruskal–Wallis) and Cramer’s V for categorical variables. Benchmarks: small = 0.01/0.1, medium = 0.06/0.3, large = 0.14/0.5, respectively. No missing data were present for the reported variables; all analyses were performed on 270 complete cases

*P-value < 0.05 was considered statistically significant

Pre-adjustment cuff pressure showed large differences among all three groups (all Bonferroni-adjusted *P* < 0.001), whereas pre-adjustment cuff volume differed primarily between the Too-low and Too-high groups (Supplementary Table S1). Adverse symptoms, including sore throat, hoarseness, and blood-tinged sputum, were uncommon and occurred at similar rates across pressure categories (all *P* > 0.05; Table 2).

### Factors associated with inappropriate cuff pressure

In exploratory multinomial logistic regression (Table 3), patient characteristics, including age, sex, and body mass index, were not associated with having cuff pressure outside the recommended range. Operation type demonstrated a significant overall association with pressure category after false-discovery-rate adjustment (global *P* = 0.034), although no individual surgical subgroup showed a significant association in multivariable analyses.

**Table 3:** Multinomial logistic regression analysis of factors associated with inappropriate endotracheal tube cuff pressure

Term	Other pressure vs Adequate pressure	Univariable analysis		Multivariable analysis			
		**OR (95%CI)**	***P*-value**^††^	**OR (95%CI)**	***P*-value**^††^	**Global** ***P*-value**^‡^	**Global** **FDR-adjusted** **P-value**^‡‡^
Age (per year)	Too-low	1.00 (0.99 – 1.02)	0.654	1.00 (0.99 – 1.02)	0.636	–	–
	Too-high	0.99 (0.97 – 1.00)	0.162	0.99 (0.97 – 1.01)	0.234	–	–
BMI (per kg/m^2^)	Too-low	0.94 (0.88 – 1.01)	0.103	–	–	–	–
	Too-high	0.99 (0.93 – 1.05)	0.741	–	–	–	–
Male (vs. Female)	Too-low	1.49 (0.83 – 2.68)	0.185	2.70 (0.82 – 8.91)	0.103	–	–
	Too-high	0.86 (0.48 – 1.53)	0.604	1.42 (0.49 – 4.08)	0.515	–	–
Non-abdominal surgery vs. Abdominal surgery							
Eye-ENT	Too-low	1.46 (0.44 – 4.85)	0.534	–	–	0.028^*^	0.034^*^
	Too-high	2.26 (0.70 – 7.30)	0.174	–	–		
Head and neck	Too-low	1.20 (0.46 – 3.15)	0.715	–	–		
	Too-high	0.88 (0.28 – 2.76)	0.826	–	–		
Urogynecologic	Too-low	0.78 (0.30 – 2.01)	0.607	–	–		
	Too-high	1.55 (0.65 – 3.71)	0.327	–	–		
Orthopedic	Too-low	1.10 (0.42 – 2.84)	0.848	–	–		
	Too-high	2.90 (1.24 – 6.79)	0.014*	–	–		
Other^†^	Too-low	1.95 (0.41 – 9.18)	0.397	–	–		
	Too-high	8.39 (2.22 – 31.65)	0.002*	–	–		
Other ETT brand vs. Covidien							
Portex	Too-low	1.07 (0.41 – 2.76)	0.894	0.93 (0.35 – 2.47)	0.890	0.030^*^	0.034^*^
	Too-high	0.26 (0.09 – 0.75)	0.013^*^	0.26 (0.09 – 0.79)	0.018^*^		
Shaoxing	Too-low	0.64 (0.29 – 1.42)	0.269	0.57 (0.26 – 1.32)	0.143		
	Too-high	0.54 (0.26 – 1.12)	0.098	0.58 (0.27 – 1.21)	0.196		
Other ETT size vs. ETT size ≤ 7.0							
ETT size 7.5	Too-low	1.45 (0.72 – 2.95)	0.298	0.70 (0.23 – 2.15)	0.531	0.316	0.240
	Too-high	1.32 (0.69 – 2.56)	0.415	1.15 (0.45 – 2.97)	0.773		
ETT size 8.0	Too-low	1.09 (0.53 – 2.23)	0.820	0.42 (0.11 – 1.66)	0.217		
	Too-high	0.53 (0.25 – 1.14)	0.104	0.41 (0.11 – 1.48)	0.176		

Data are presented as odds ratios (OR) with 95% confidence intervals (CI)

*ETT* Endotracheal tube, *BMI* Body mass index, *ENT* Ear, nose, and throat

^†^Other surgery includes radiologic, neurosurgical, endoscopic, and plastic procedures

^††^Wald tests were used to derive *P*-values for univariable and multivariable models. For multi-category predictors, ‡ global P-values were estimated using likelihood ratio tests (LRT) and ‡‡ subsequently adjusted for multiple comparisons via the Benjamini–Hochberg false discovery rate (FDR) method

^*^P < 0.05 was considered statistically significant. Interpretation of device-level associations (e.g., ETT brand) should consider the absence of detailed provider-level data, as described in the Limitations section

ETT brand was significantly associated with cuff-pressure status (global *P* = 0.034). Compared with Covidien tubes, Portex tubes were associated with lower odds of being in the Too-high rather than Adequate cuff-pressure category (adjusted OR 0.26, 95% CI 0.09 – 0.79). ETT internal diameter was not significantly associated with cuff-pressure adequacy.

### Relationship between inflation volume and achieved pressure

Generalized additive modelling demonstrated a modest but statistically significant nonlinear association between inflation volume and final cuff pressure (smooth term *P* = 0.003). However, the overall relationship was weak, with minimal linear correlation (Pearson r = −0.11; Spearman ρ = −0.18) (Supplementary Figure S1A; Supplementary Table S2). The spline fit showed shallow curvature and wide scatter, indicating limited discriminative value of inflation volume. Stratified plots by ETT size and brand showed similarly variable patterns with substantial within-group dispersion (Supplementary Figure S1B).

### Prediction models: cuff inflation volume required for adequate pressure

A multivariable linear regression model was developed to estimate inflation volume required to achieve a cuff pressure of 20 – 30 cmH₂O (Table 4). Age, sex, ETT internal diameter, and ETT brand collectively explained 33.6% of the variance (R^2^ = 0.336; RMSE = 1.19 mL). Greater age, male sex, and use of Portex or Shaoxing tubes were associated with higher inflation volumes.

**Table 4:** Linear regression model for predicting cuff inflation volume (mL), aiming for adequate cuff pressure (20 – 30 cm H_2_O)

Variable	β Estimate	Standard Error	95% CI	*P*-value
Intercept	3.663	0.292	(3.093 – 4.234)	< 0.001^*^
Age (per year)	0.021	0.004	(0.013 – 0.030)	< 0.001^*^
Male (vs. Female)	1.040	0.268	(0.512 – 1.567)	< 0.001^*^
ETT size 7.5 mm (vs. ≤ 7.0 mm)	0.159	0.249	(−0.331 – 0.649)	0.523
ETT size 8.0 mm (vs. ≤ 7.0 mm)	0.504	0.317	(−0.120 – 1.129)	0.113
ETT Brand: Portex (vs. Covidien)	0.651	0.243	(0.173 – 1.129)	0.008^*^
ETT Brand: Shaoxing (vs. Covidien)	0.310	0.187	(−0.059 – 0.678)	0.099

A multivariable linear regression model was developed to predict the final endotracheal tube (ETT) cuff inflation volume (mL) using readily measurable patient and device factors. The final model included age, sex, ETT internal diameter (mm ID), and ETT brand, explaining 33.6% of the variance in observed cuff volume (*R*^2^ = 0.336; RMSE = 1.19 mL; *n =* 270)

Regression coefficients (β) represent the expected change in cuff inflation volume (mL) per one-unit increase in the corresponding predictor, holding other variables constant. Reference categories: Female sex; ETT size ≤ 7.0 mm ID; ETT brand = Covidien

*R*^2^ coefficient of determination, *RMSE* Root-mean-square error, *CI* Confidence interval, *ETT* Endotracheal tube

**Fig. 4:**
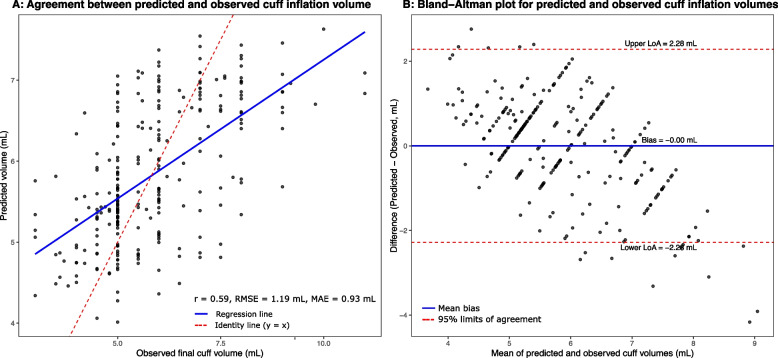
Agreement between predicted and observed cuff inflation volumes. **A** Correlation between predicted and observed final cuff inflation volumes. Each point represents one observation. The blue line indicates the regression fit, and the red dashed line represents the identity line (y = x). The correlation coefficient (r = 0.59), root mean square error (RMSE = 1.19 mL) and mean absolute error (MAE = 0.93 mL) are shown. **B** Bland–Altman plot showing the mean bias (solid blue line) and 95% limits of agreement (mean ± 1.96 SD; dashed red lines; upper limit +2.28 mL, lower limit −2.28 mL)

Agreement between predicted and observed volumes was moderate. The correlation plot showed r = 0.59 (Fig. 4A), and Bland–Altman analysis demonstrated near-zero mean bias with acceptable limits of agreement (Fig. 4B). Regression diagnostics revealed no major violations (Supplementary Figure S2).

**Table:** 

Predicted cuff inflation volume (mL)
Predicted cuff volume (mL) = 3.663 + (0.021×Age [years]) + (1.040×Male) + ($\beta_\mathrm{ETT\;size}$) + ($\beta_\mathrm{ETT\;brand}$)
where
$$\begin{array}{ll}\beta_\mathrm{ETT\;size} &= 0\;\mathrm{for\;size} \le 7\;\mathrm{mm} \left(\mathrm{Reference}\right)\\&+0.159\;\mathrm{for\;size}\;7.5\;\mathrm{mm}\\&+0.504\;\mathrm{for\;size}\;8.0\;\mathrm{mm}\\\beta_\mathrm{ETT\;brand} &= 0\;\mathrm{for\;Covidien} \left(\mathrm{Reference}\right)\\&+0.310\;\mathrm{for\;Shaoxing}\\&+0.651\;\mathrm{for\;Portex}\end{array}$$
This linear model estimates the final cuff inflation volume (mL) based on simple patient and device characteristics.
• Age = patient’s age in years
• Male = 1 for male, 0 for female (reference)
• ETT size = endotracheal tube internal diameter (mm), with ETT size ≤ 7.0 mm as the reference
• ETT brand = Portex or Shaoxing, with Covidien as the reference

### Prediction models: predicting final cuff pressure

In exploratory analyses, the association between inflation volume and final cuff pressure remained weak. The generalized additive model showed a modest nonlinear effect (edf 2.5; *P* = 0.003) but explained limited variability (Supplementary Figure S3). A corresponding linear regression model accounted for only 5.7% of the variance (R^2^ = 0.057) and did not identify significant associations for volume or other covariates (Supplementary Table S3). Model assumptions were adequately met (Supplementary Figure S4).

Agreement between predicted and observed cuff pressure was poor, with wide dispersion in prediction–observation plots (Supplementary Figure S5A) and broad Bland–Altman limits of agreement (–6.14 to +6.14 cmH₂O) despite minimal mean bias (Supplementary Figure S5B). The full prediction equation appears in Supplementary Table S3.

## Discussion

Our prevalence of adequate cuff pressure (39.6%) is slightly higher than most previous reports from routine practice without manometric guidance, where only about 25 – 30% of patients were within the 20 – 30 cmH₂O range and 60 – 75% had inappropriate pressures. Studies by Sengupta et al. [3], Shamsi et al. [23], and Shibasaki et al. [24] consistently found that around half of patients were overinflated and 10 – 55% were underinflated, with only 27 – 39% in the target range. Mean cuff pressures in these cohorts clustered above 30 cmH₂O (often around 35 cmH₂O with wide variability), indicating a strong tendency towards overinflation. Against this background, our finding that 60.4% of patients had inappropriate cuff pressure confirms that the problem remains common in contemporary practice.

The somewhat lower proportion of overinflation in our cohort compared with some earlier studies may reflect local practice. In our institution, inflation is typically initiated with approximately 5 mL of air and then adjusted clinically (e.g. according to audible leak), whereas Sengupta et al.[3] reported lower initial volumes (around 2.5 – 2.9 mL). Starting at a modest fixed volume may reduce extreme overinflation, but our data show that this strategy still leaves more than half of patients outside the recommended pressure range, with both too-low and too-high pressures occurring frequently. Empirical adjustment based on leak or pilot-balloon palpation is therefore insufficient to ensure safe cuff pressure.

Although international guidelines recommend manometric monitoring, real-world data show that cuff manometers are used infrequently. Surveys from Nigeria reported that only a small minority of airway care providers had ever used a tracheal cuff manometer and that manometers were neither available nor used in surveyed tertiary hospitals [25]. In a bi-national survey of anaesthetists in Australia and New Zealand, 78% reported ready access to a cuff manometer, but only 40% used one routinely [26]. Sengupta et al. [3] similarly noted that manometers, although the simplest device for measuring cuff pressure, were “not widely available” in their U.S. hospitals. In our own setting, intraoperative manometry is also not used routinely despite availability in some operating rooms. These observations explain why subjective techniques remain the dominant methods worldwide and why inappropriate cuff pressure is so prevalent.

To explore this gap, we developed a prediction model for initial cuff inflation volume using readily available clinical variables, achieving an R^2^ of 0.34. This performance is reasonable for simple predictors but modest compared with the work by Shibasaki et al. [24], who linked optimal cuff volume to radiographic tracheal diameter (R^2^ = 0.83) and achieved target pressures in about 65% of patients, versus 28% with manual palpation. A second model based on height and age achieved more moderate performance (adjusted R^2^ = 0.44) and target pressures in approximately 45% of patients. Notably, even their best formula still failed in about one-third of patients, and the most accurate approach required preoperative chest radiography and precise tracheal measurements, which are not routinely obtained and may vary with technique, limiting broad applicability.

By contrast, our model relies only on routinely available clinical information and ETT characteristics. To illustrate the limited explanatory value of such variables, model coefficients were examined to assess the direction and relative magnitude of their associations with inflation volume. These exploratory patterns are presented for interpretative purposes only and are not intended to guide clinical practice or replace direct cuff-pressure measurement.

Consistent with the poor performance of models predicting final cuff pressure and the substantial inter-individual variability observed, our data do not support the use of any volume-based formula as a substitute for manometric monitoring. Rather, these findings align with prior modelling research in demonstrating the inherent limitations of empirical and volume-based estimation strategies.

The inability of regression-based models to identify robust predictors reflects substantial inter-individual variability in cuff pressure behavior and demonstrates the inherent limitations of volume-based estimation strategies.

### Strengths and limitations

Strengths of this study include its prospective design, use of standardised manometric cuff-pressure measurements in a real-world operating theatre environment, and evaluation of both patient-level and device-level determinants. We explicitly distinguished between under- and overinflation and quantified the prevalence of inappropriate cuff pressure in a mixed adult surgical population. By constructing and testing models for both cuff inflation volume and final pressure, we provide quantitative evidence on how far simple clinical variables can support cuff management and where their limitations lie.

Several limitations should be acknowledged. First, this was a single-centre study in an academic hospital, which may limit generalisability to other institutions with different case mixes, devices, and practice patterns. Second, cuff pressure was measured at a single early intraoperative time point; we did not perform serial measurements to capture changes related to repositioning, ventilation adjustments, nitrous oxide use, or longer anaesthetic duration. Third, we did not formally assess inter-rater reliability for cuff pressure measurement; however, all measurements and adjustments were performed with a single calibrated device using a standardised written protocol, which likely minimised measurement error. Fourth, we did not collect detailed data on anaesthesia provider experience level or intubation difficulty, and these provider-related factors may influence initial cuff inflation practices. Fifth, ETT brand allocation was not randomised and reflected institutional procurement, so residual confounding by unmeasured factors cannot be excluded. Sixth, postoperative airway symptoms were assessed at 24 h and may underestimate subclinical injury and longer-term outcomes such as tracheal stenosis or pneumonia. Finally, some subgroup analyses were underpowered, and we did not perform external validation of the prediction model in an independent cohort, which is planned for a subsequent phase of this research. Given the exploratory nature of the secondary analyses and the sample size, residual risks of type I error inflation and model overfitting cannot be fully excluded; accordingly, findings from the regression analyses should be interpreted as descriptive and hypothesis-generating rather than confirmatory. Formal model-stability procedures were not undertaken, as the modelling was not intended for prediction or confirmation but to illustrate the limited explanatory value of routinely available clinical variables.

### Clinical implications

Clinically, our findings emphasise that ETT cuff pressure cannot be safely managed by palpation or fixed inflation volumes alone. Even in an experienced centre, almost two-thirds of patients were outside the 20 – 30 cmH₂O range, and both under- and overinflation were common. Because patient characteristics were not helpful predictors and cuff pressure appeared to be influenced by ETT brand, applying a single “standard” volume across different tubes is unlikely to be safe. Our exploratory prediction model may offer insight into the systematic variability in cuff inflation volume related to patient and device characteristics. However, these findings are illustrative only and were not developed or validated for clinical decision-making. They should not be interpreted as providing guidance for cuff inflation in routine practice and do not remove the need for direct cuff-pressure measurement.

In practice, several barriers likely contribute to the low uptake of routine cuff-pressure monitoring, including time constraints, lack of integration into existing safety checklists, limited availability of devices, and insufficient training in cuff management. Potential implementation strategies include embedding cuff-pressure measurement into perioperative checklists (such as the WHO Surgical Safety Checklist [27]), ensuring that an affordable manometer is available at each anaesthesia workstation, incorporating cuff management into simulation-based or competency-based training, and, where feasible, using prompts or electronic systems to support and document cuff-pressure measurements.

Taken together with previous work, these results support routine use of a cuff manometer to set and periodically check ETT cuff pressure, including in resource-limited settings where low-cost intermittent manometers and simple checklist-based prompts may be particularly valuable. Where available, structured monitoring protocols or automated cuff-pressure control systems may further improve pressure stability, and future studies should examine their effects on longer-term airway and pulmonary outcomes.

## Conclusion

In adult surgical patients undergoing elective procedures with endotracheal intubation, inappropriate ETT cuff pressure is common when inflation is performed empirically, with substantial proportions of both under- and overinflation. Patient characteristics are poor predictors of cuff-pressure deviations, whereas ETT brand has a meaningful influence. Simple clinical variables and cuff inflation volume explain only moderate variability in the volume required for adequate pressure and are poor predictors of final cuff pressure. These findings support routine manometric monitoring as a key patient-safety measure and caution against relying on volume-based inflation alone. Future multicentre studies, particularly in diverse healthcare systems, should evaluate standardised cuff-pressure monitoring strategies and their impact on longer-term airway and pulmonary outcomes.

## Supplementary Information

Supplementary Material 1.

## Data Availability

The datasets generated and analysed during the current study are available from the corresponding author on reasonable request. De-identified data that support the findings of this study can be shared with qualified investigators for academic research purposes after approval of a formal data-sharing agreement. No publicly archived datasets are associated with this study.

## Abbreviations

AIC: Akaike information criterion
BIC: Bayesian information criterion
BMI: Body mass index
CI: Confidence interval
cmH₂O: Centimetres of water
ENT: Ear, nose, and throat
ETT: Endotracheal tube
FDR: False discovery rate
GAM: Generalized additive model
ICU: Intensive care unit
ID: Internal diameter
IQR: Interquartile range
MAE: Mean absolute error
OR: Odds ratio
PEEP: Positive end-expiratory pressure
PIP: Peak inspiratory pressure
PVC: Polyvinyl chloride
R^2^: Coefficient of determination
RMSE: Root-mean-square error
Δ: Change (difference between post- and pre-adjustment value)
