# Supplementary material for: Prevalence and determinants of inappropriate endotracheal cuff pressure in adult surgical patients undergoing general anesthesia: a prospective observational study
Source: BMC Anesthesiol. 2026 Mar 12;26:249. doi: 10.1186/s12871-026-03738-7 (PMC13094173; doi:10.1186/s12871-026-03738-7)

Supplementary Appendix

Supplementary Appendix I *Page2-4*

STROBE checklist of items that should be included in reports of cohort studies

Supplementary Appendix II

Supplementary Table S1 *Page 5*

Pairwise comparisons of pre- and post-adjustment cuff parameters among pressure groups after Bonferroni adjustment.

Supplementary Table S2 *Page 6*

Generalized additive model for the relationship between endotracheal tube cuff inflation volume and final cuff pressure.

Supplementary Table S3 *Page 7-8*

Linear regression model for predicting final endotracheal tube (ETT) cuff pressure (cmH_2O) from inflation volume and covariates.

Supplementary Figure S1. *Page 9*

Relationship between cuff inflation volume and measured pressure.

Supplementary Figure S2. *Page 10*

Regression diagnostics for the cuff inflation volume prediction model.

Supplementary Figure S3. *Page 11*

Smooth function of cuff inflation volume from the generalized additive model (GAM).

Supplementary Figure S4 *Page 12*

Model diagnostic plots for the linear regression predicting endotracheal tube cuff pressure.

Supplementary Figure S5 *Page 13*

Agreement between predicted and observed endotracheal tube cuff pressure.

Supplementary Appendix I.

STROBE checklist of items that should be included in reports of cohort studies.

	Item No	Recommendation	
**Title and abstract**	1	(*a*) Indicate the study's design with a commonly used term in the title or the abstract	*Page 1*
		(*b*) Provide in the abstract an informative and balanced summary of what was done and what was found	*Page 2-3*
**Introduction**			
Background/rationale	2	Explain the scientific background and rationale for the investigation being reported	*Page 3-4*
Objectives	3	State specific objectives, including any prespecified hypotheses	*Page 4-5*
**Methods**			
Study design	4	Present key elements of study design early in the paper	*Page 5*
Setting	5	Describe the setting, locations, and relevant dates, including periods of recruitment, exposure, follow-up, and data collection	*Page 5-8*
Participants	6	(*a*) Give the eligibility criteria, and the sources and methods of selection of participants. Describe methods of follow-up	*Page 5-8*
		(*b*) For matched studies, give matching criteria and number of exposed and unexposed	*Not applicable*
Variables	7	Clearly define all outcomes, exposures, predictors, potential confounders, and effect modifiers. Give diagnostic criteria, if applicable	*Page 8*
Data sources/ measurement	8*	For each variable of interest, give sources of data and details of methods of assessment (measurement). Describe comparability of assessment methods if there is more than one group	*Page 6-8*
Bias	9	Describe any efforts to address potential sources of bias	*Page 6-7, 17-18*
Study size	10	Explain how the study size was arrived at	*Page 9*
Quantitative variables	11	Explain how quantitative variables were handled in the analyses. If applicable, describe which groupings were chosen and why	*Page 6-10*
Statistical methods	12	(*a*) Describe all statistical methods, including those used to control for confounding	*Page 9-10*
		(*b*) Describe any methods used to examine subgroups and interactions	*Not applicable*
		(*c*) Explain how missing data were addressed	*Page 9*

		(*d*) If applicable, explain how loss to follow-up was addressed	*Page 10, Figure 1.*
		(*e*) Describe any sensitivity analyses	*Not applicable*
**Results**			
Participants	13*	(a) Report numbers of individuals at each stage of study—eg numbers potentially eligible, examined for eligibility, confirmed eligible, included in the study, completing follow-up, and analysed	*Page 10, Figure 1.*
		(b) Give reasons for non-participation at each stage	*Page 10, Figure 1.*
		(c) Consider use of a flow diagram	*Figure 1.*
Descriptive data	14*	(a) Give characteristics of study participants (eg demographic, clinical, social) and information on exposures and potential confounders	*Page 11-12, Table 1.*
		(b) Indicate number of participants with missing data for each variable of interest	*Page 9, Table1-2*
		(c) Summarise follow-up time (eg, average and total amount)	*Page 8*
Outcome data	15*	Report numbers of outcome events or summary measures over time	*Page 10-12, Table 2, Figure 2-3*
Main results	16	(*a*) Give unadjusted estimates and, if applicable, confounder-adjusted estimates and their precision (eg, 95% confidence interval). Make clear which confounders were adjusted for and why they were included	*Table 1-3, Supplementary Tables (S1–S3)*
		(*b*) Report category boundaries when continuous variables were categorized	*Page 8*
		(*c*) If relevant, consider translating estimates of relative risk into absolute risk for a meaningful time period	*Not applicable*
Other analyses	17	Report other analyses done—eg analyses of subgroups and interactions, and sensitivity analyses	*Page 13-14*
**Discussion**			
Key results	18	Summarise key results with reference to study objectives	*Page 14*
Limitations	19	Discuss limitations of the study, taking into account sources of potential bias or imprecision. Discuss both direction and magnitude of any potential bias	*Page 17-18*
Interpretation	20	Give a cautious overall interpretation of results considering objectives, limitations, multiplicity of analyses, results from similar studies, and other relevant evidence	*Page 14-16*
Generalisability	21	Discuss the generalisability (external validity) of the study results	*Page 17-18*
**Other information**			

Funding	22	Give the source of funding and the role of the funders for the present study and, if applicable, for the original study on which the present article is based	*Page 21*

*Give information separately for exposed and unexposed groups.

Note: An Explanation and Elaboration article discusses each checklist item and gives methodological background and published examples of transparent reporting. The STROBE checklist is best used in conjunction with this article (freely available on the Web sites of PLoS Medicine at http://www.plosmedicine.org/, Annals of Internal Medicine at http://www.annals.org/, and Epidemiology at http://www.epidem.com/). Information on the STROBE Initiative is available at http://www.strobe-statement.org.

Supplementary Table S1. Pairwise comparisons of pre- and post-adjustment cuff parameters among pressure groups after Bonferroni adjustment.

Parameters	Comparison	P-value
Pre-adjustment cuff volume, mL	Too low vs. Adequate	0.063
	Too high vs. Adequate	0.177
	Too high vs. Too low	0.001*
Post-adjustment ETT cuff volume, mL	Too low vs. Adequate	< 0.001*
	Too high vs. Adequate	< 0.001*
	Too high vs. Too low	< 0.001*
Pre-adjustment cuff pressure, cmH_2O	Too low vs. Adequate	< 0.001*
	Too high vs. Adequate	< 0.001*
	Too high vs. Too low	< 0.001*
Post-adjustment cuff pressure, cmH_2O	Too low vs. Adequate	0.610
	Too high vs. Adequate	< 0.001*
	Too high vs. Too low	< 0.001*
Pre-adjustment PIP, cmH_2O	Too low vs. Adequate	0.047*
	Too high vs. Adequate	0.232
	Too high vs. Too low	> 0.999

Data represent pairwise P-values obtained from Bonferroni-adjusted Wilcoxon rank-sum tests among the Too low, Adequate, and Too high pressure groups. * Statistical significance was defined as P-value <0.05 after adjustment. Abbreviations: ETT, endotracheal tube; cmH_2O, centimeters of water; PIP, peak inspiratory pressure; Δ, delta (change from pre- to post-adjustment value); vs., versus; PIP, peak inspiratory pressure.

Supplementary Table S2. Generalized additive model for the relationship between endotracheal tube cuff inflation volume and final cuff pressure

Variable	β Estimate	Standard Error	95% CI	P-value
**Intercept**	26.45	0.79	(24.893, 27.999)	< 0.001*
Age (per year)	−0.011	0.012	(−0.035, 0.013)	0.374
Male (vs. Female)	+1.14	0.72	(−0.283, 2.559)	0.118
ETT size 7.5 mm (vs. ≤ 7.0 mm)	+0.08	0.65	(−1.201, 1.364)	0.901
ETT size 8.0 mm (vs. ≤ 7.0 mm)	−0.84	0.84	(−2.479, 0.801)	0.317
ETT Brand: Portex (vs. Covidien)	−0.69	0.50	(−1.657, 0.286)	0.168
ETT Brand: Shaoxing (vs. Covidien)	−1.27	0.53	(−2.303, −0.236)	0.017*

A generalized additive model (GAM) with a smooth spline term for final air volume was fitted to evaluate non-linear effects on final cuff pressure (Gaussian family, identity link). The smooth term (*edf* = 2.50, $F = 4.65$, $p = 0.003$) indicated a modest non-linear volume–pressure relationship. Model performance: $R^2 = 0.102$; RMSE = 3.05 cmH_2O; MAE = 2.57 cmH_2O; $n = 270$. Reference categories: female sex; ETT size ≤ 7.0 mm; ETT brand = Covidien.

Model comparison with a linear specification (*Final cuff pressure ~ Final air volume + covariates*) showed that adding spline terms modestly improved fit ($F = 7.49$, $p = 0.0007$; ΔAIC = –11.2; ΔBIC = –4.0), confirming a mild non-linear effect of cuff volume.

Abbreviations: ETT = endotracheal tube; *edf* = effective degrees of freedom; CI = confidence interval; SE = standard error; RMSE = root-mean-square error; MAE = mean absolute error.

Supplementary Table S3. Linear regression model for predicting final endotracheal tube (ETT) cuff pressure (cmH_2O) from inflation volume and covariates

Variable	β Estimate	Standard Error	95% CI	P-value
**Intercept**	27.591	0.990	(25.643, 29.539)	< 0.001 *
Final air volume (mL)	−0.279	0.166	(−0.605, 0.046)	0.093 ·
Age (per year)	−0.014	0.012	(−0.038, 0.010)	0.258
Male (vs. Female)	1.080	0.740	(−0.378, 2.538)	0.146
ETT size 7.5 mm (vs. ≤ 7.0 mm)	0.075	0.668	(−1.239, 1.389)	0.911
ETT size 8.0 mm (vs. ≤ 7.0 mm)	−0.950	0.854	(−2.628, 0.728)	0.267
ETT Brand: Portex (vs. Covidien)	−0.501	0.661	(−1.803, 0.801)	0.450
ETT Brand: Shaoxing (vs. Covidien)	0.706	0.505	(−0.284, 1.696)	0.163

A multivariable linear regression model was developed to estimate the final endotracheal tube (ETT) cuff pressure (cm H_2O) from the inflation volume (mL) and patient/device covariates. The model explained 5.7 % of the variance ($R^2 = 0.057$; adjusted $R^2 = 0.032$; residual SE = 3.18 cm H_2O; n = 270). Cross-validation using repeated 10-fold CV yielded mean RMSE = 3.21 cm H_2O, MAE = 2.72 cm H_2O, and mean $R^2 = 0.05$. Reference categories: female sex; ETT size ≤ 7.0 mm; ETT brand = Covidien.

Abbreviations: ETT = endotracheal tube; CI = confidence interval; SE = standard error; RMSE = root-mean-square error; MAE = mean absolute error.

Prediction equation

$$\text{Predicted cuff pressure} = 27.591 - (0.279 \times \text{Volume [mL]}) - (0.014 \times \text{Age [years]})$$

$$+ (1.080 \times \text{Male}) + \beta_{\text{ETT size}} + \beta_{\text{ETT brand}}$$

where

$\beta_{\text{ETT size}}$ = 0 for size ≤ 7 mm (reference)

+0.075 for size 7.5 mm

−0.950 for size 8.0 mm

$\beta_{\text{ETT brand}}$ = 0 for Covidien (reference)

−0.501 for Portex

+0.706 for Shaoxing

This linear model estimates the predicted endotracheal tube (ETT) cuff pressure (cmH_2O) based on simple patient and device characteristics, with the inflated cuff volume.

- **Volume** = Volume inflation in ETT cuff (mL)
- **Age** = patient's age in years
- **Male** = 1 for male, 0 for female (reference)
- **ETT size** = endotracheal tube internal diameter (mm), with ETT size ≤ 7.0 mm as the reference
- **ETT brand** = Portex or Shaoxing, with Covidien as the reference

Supplementary Figure S1. Relationship between cuff inflation volume and measured pressure.

(A) Overall scatter plot showing linear regression (solid blue) and LOESS fit (dashed red) lines for the association between final cuff volume and final cuff pressure. The correlation was weak (Pearson r = −0.11; Spearman ρ = −0.18), indicating that inflation volume alone poorly predicts the achieved cuff pressure.

(B) Volume–pressure patterns stratified by endotracheal tube (ETT) size and brand. While the general trend suggests a nonlinear relationship, substantial variability was observed within each subgroup, underscoring that target cuff pressure cannot be reliably estimated from inflation volume or tube characteristics alone. Minor gridlines were removed and light major grids retained for visual clarity.

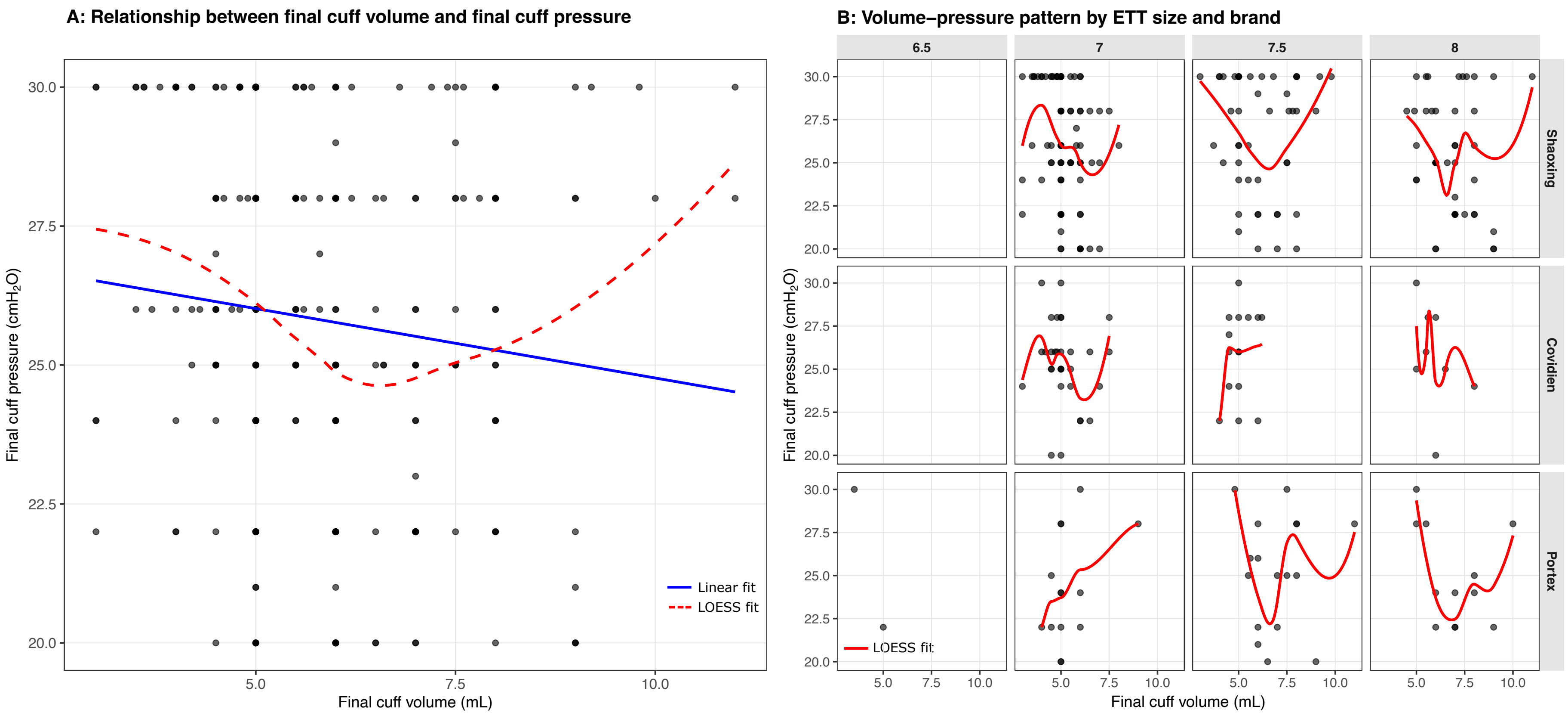

Supplementary Figure S2. Regression diagnostics for the cuff inflation volume prediction model

Diagnostic plots assessing linear model assumptions and influential observations for the cuff inflation volume prediction model.

(A) *Residuals vs Fitted*: evaluates linearity and detects patterns suggesting model mis-specification;

(B) *Normal Q–Q plot*: assesses normality of residuals;

(C) *Scale–Location plot*: checks homoscedasticity;

(D) *Residuals vs Leverage*: identifies influential data points using Cook's distance.

Overall, no major violations of linear model assumptions were observed.

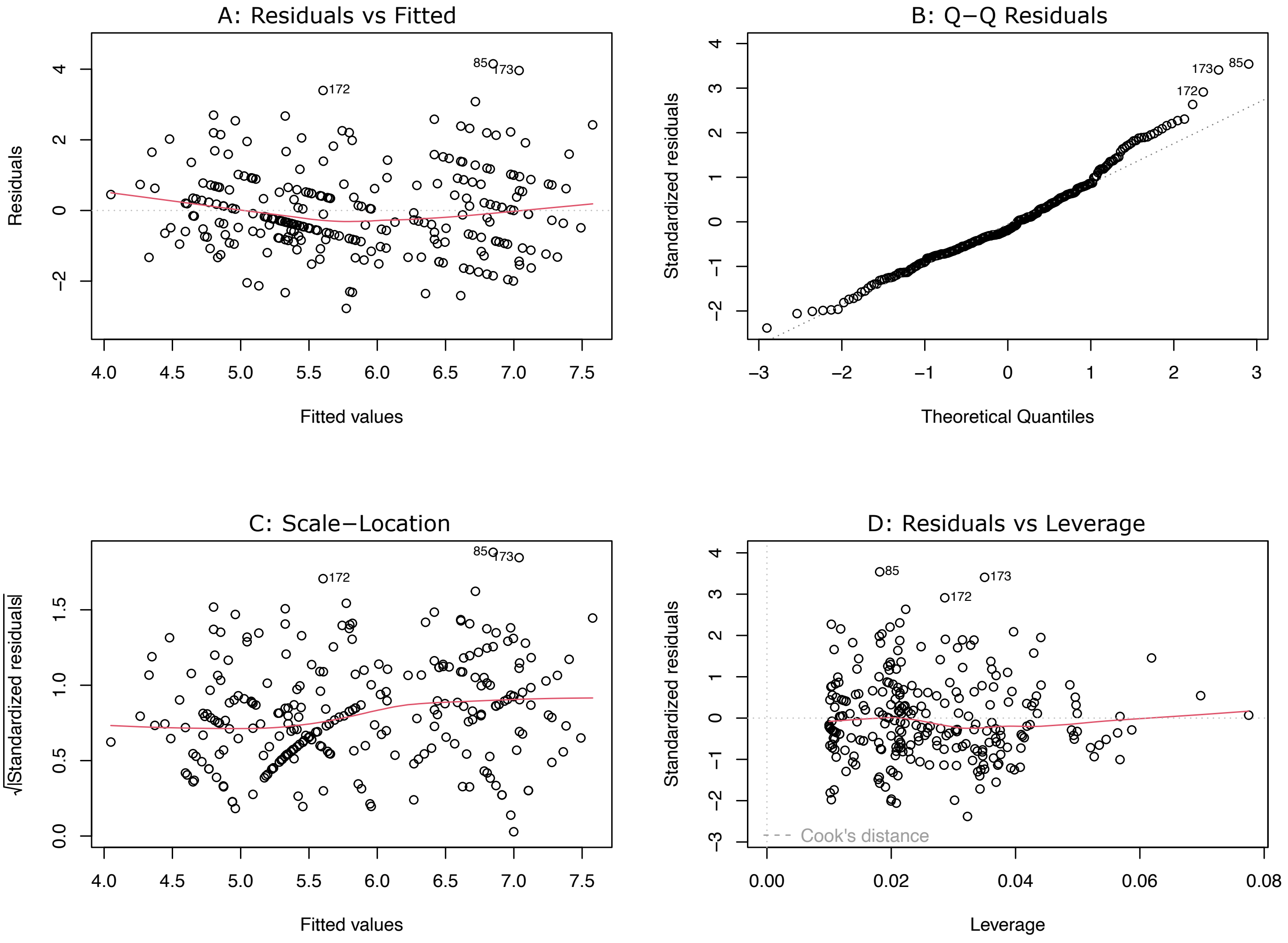

Supplementary Figure S3. Smooth function of cuff inflation volume from the generalized additive model (GAM).

Partial effect of final cuff inflation volume (mL) on final cuff pressure is shown as a penalized regression spline (solid line) with 95% confidence band (shaded area), derived from a GAM adjusted for age, sex, ETT size group, and ETT brand. Dots represent partial residuals, and tick marks along the x-axis indicate the distribution of cuff inflation volumes. The spline term was statistically significant (effective degrees of freedom = 2.5; F = 4.65; p = 0.003), indicating a modest non-linear volume–pressure relationship.

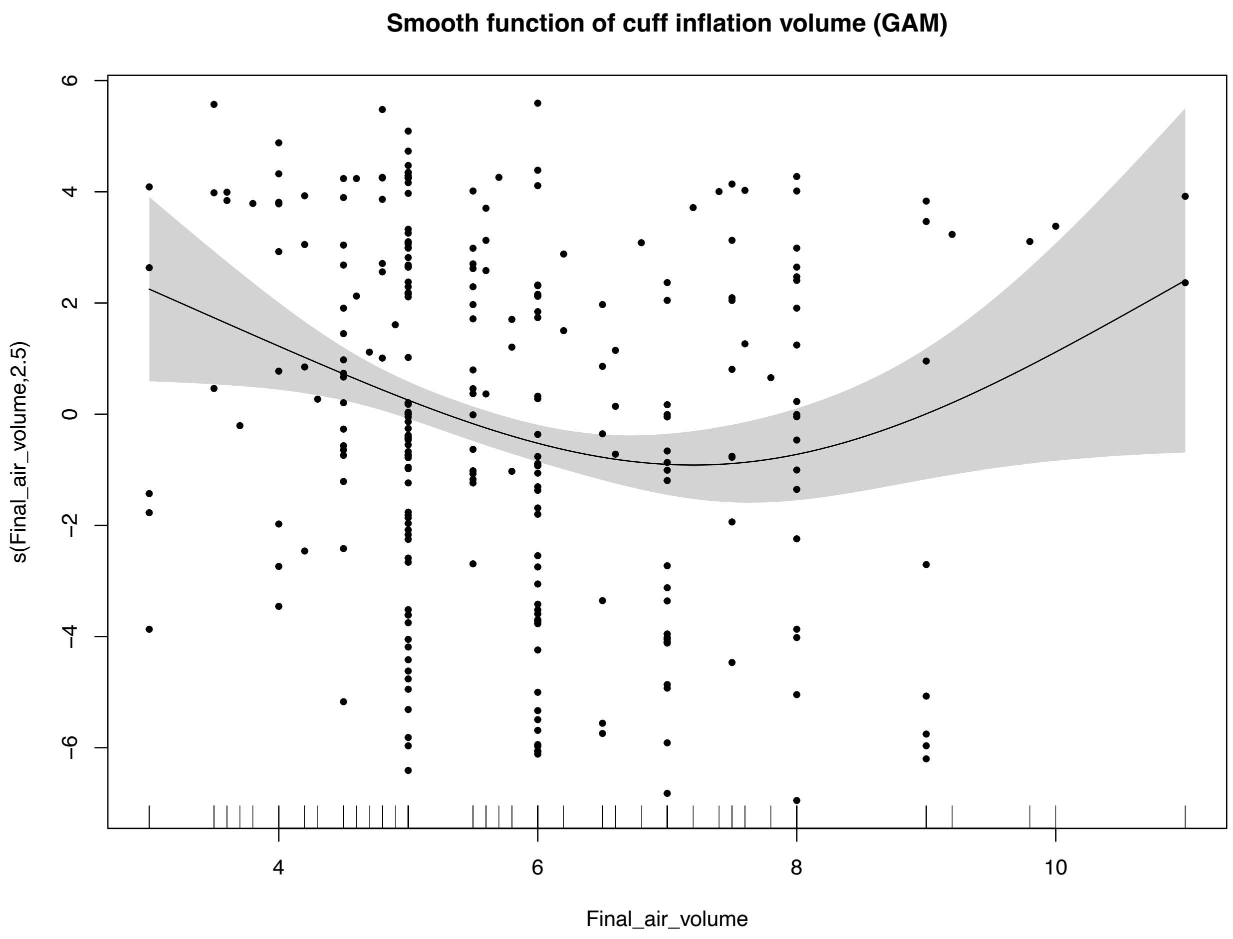

Supplementary Figure S4. Model diagnostic plots for the linear regression predicting endotracheal tube cuff pressure

Diagnostic assessments of the linear regression model are shown.

(A) Residuals versus fitted values demonstrates no major violation of linearity, with only mild structure reflecting the limited explanatory capacity of the model.

(B) Normal Q–Q plot indicates moderate deviation from normality at the distribution tails.

(C) Scale–location plot shows broadly homogeneous variance across fitted values, with minor heteroscedasticity.

(D) Residuals versus leverage identifies a small number of higher-leverage observations, none exceeding Cook's distance thresholds, indicating no unduly influential cases.

Overall, these diagnostics support the stability of the fitted model, while indicating expected limitations related to the low proportion of variance explained.

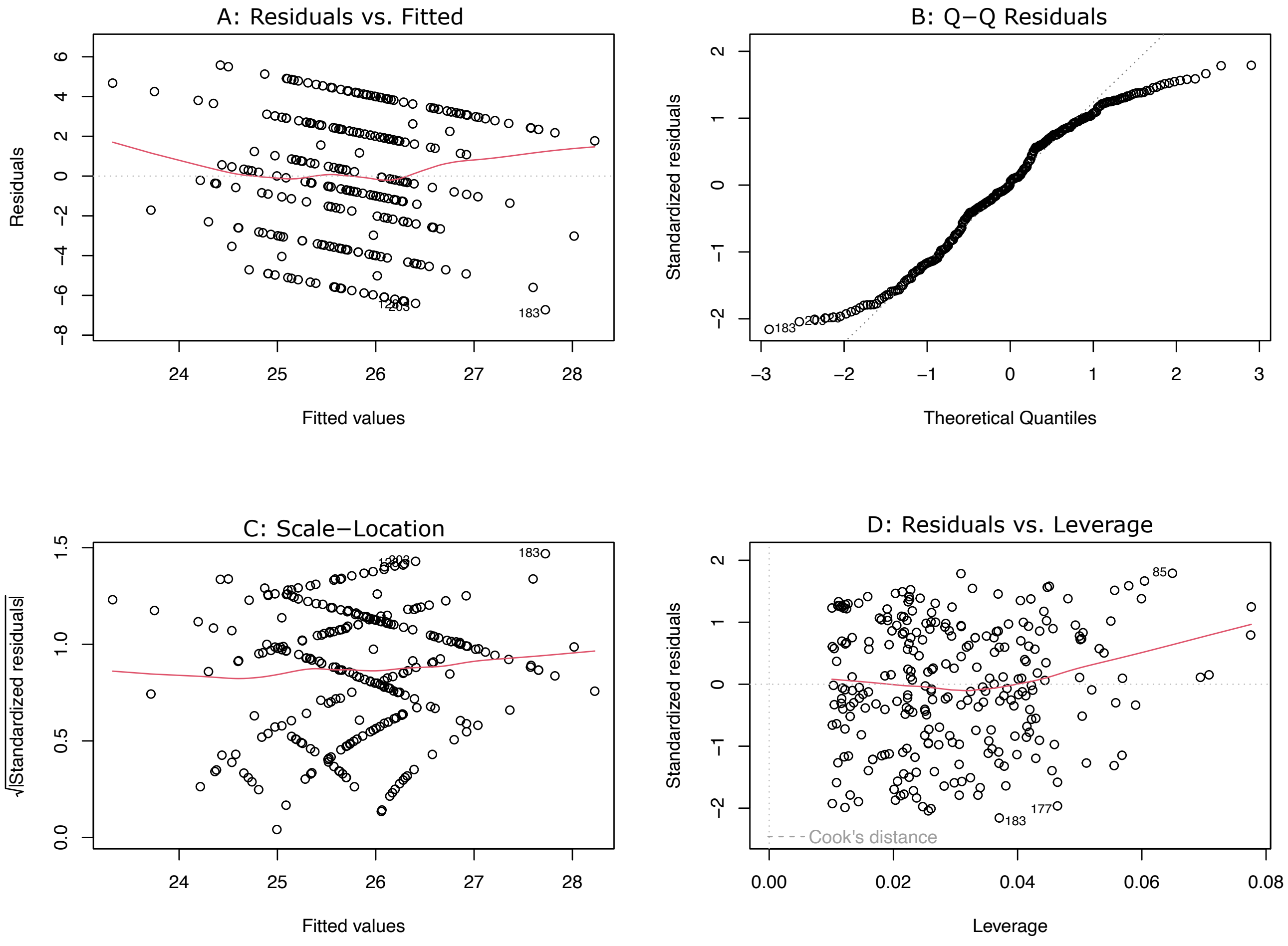

Supplementary Figure S5. Agreement between predicted and observed endotracheal tube cuff pressure

(A) Agreement plot comparing **predicted** versus **observed** cuff pressure. The blue line represents the fitted regression line, and the red dashed line shows the identity line (*y* = *x*). Model performance statistics are displayed (R^2 = 0.050, RMSE = 3.21 cmH_2O, MAE = 2.72 cmH_2O), indicating limited predictive accuracy and substantial unexplained variability.

(B) Bland–Altman plot evaluating agreement between predicted and observed cuff pressure. The solid blue line denotes the mean bias (–0.00 cmH_2O), and the red dashed lines indicate the 95% limits of agreement (–6.14 to 6.14 cmH_2O). The wide limits of agreement show considerable individual-level disagreement between measured and predicted values.

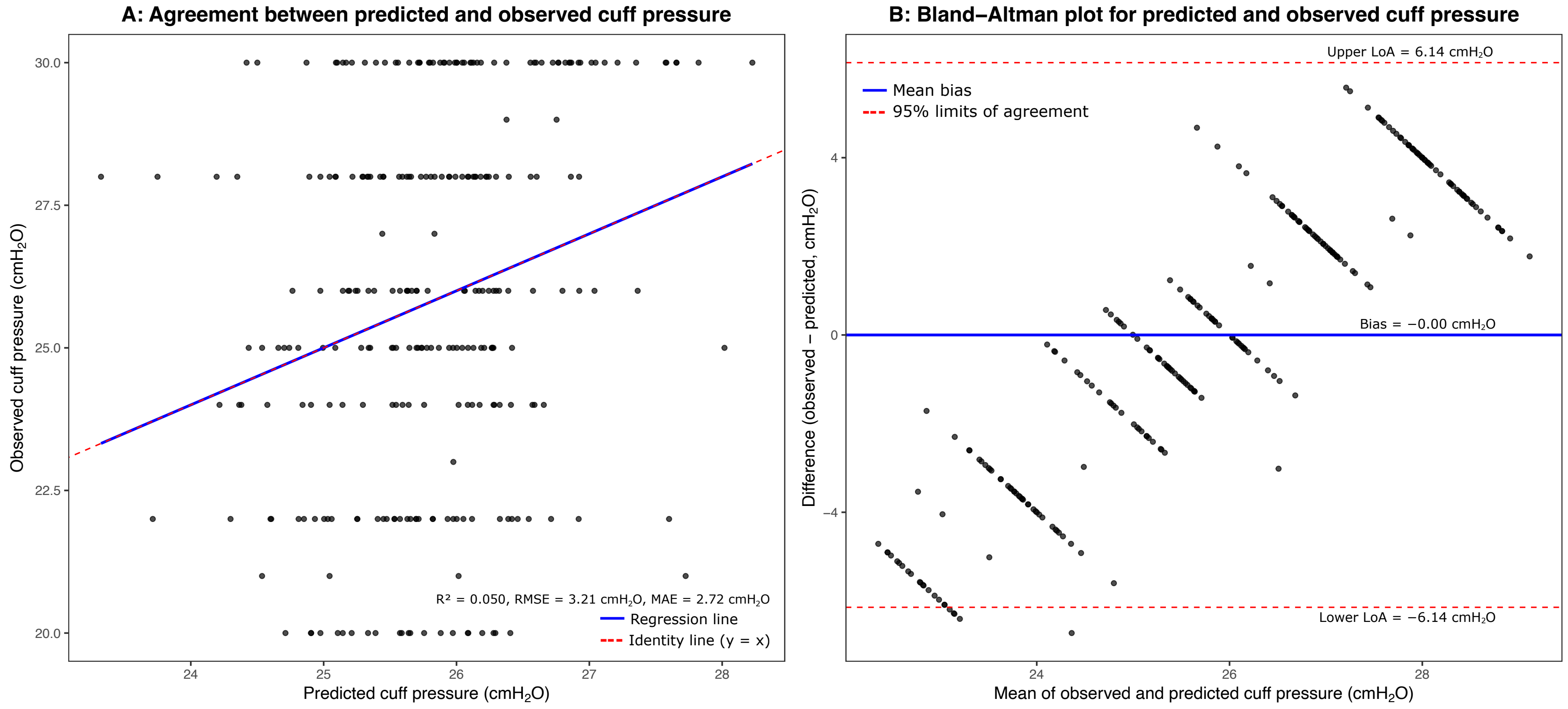

Supplement: Supplementary file 1 — Supplementary Material 1. [file 12871_2026_3738_MOESM1_ESM.pdf]
